# Dietary Lipids Affect the Onset of Hibernation in the Garden Dormouse (*Eliomys quercinus*): Implications for Cardiac Function

**DOI:** 10.3389/fphys.2018.01235

**Published:** 2018-09-18

**Authors:** Sylvain Giroud, Gabrielle Stalder, Hanno Gerritsmann, Anna Kübber-Heiss, Jae Kwak, Walter Arnold, Thomas Ruf

**Affiliations:** Department of Integrative Biology and Evolution, Research Institute of Wildlife Ecology, University of Veterinary Medicine, Vienna, Austria

**Keywords:** fatty acids, PUFA, linoleic acid, docosahexaenoic acid, torpor, hibernation, insectivorous

## Abstract

Dietary lipids strongly influence patterns of hibernation in heterotherms. Increased dietary uptake of n-6 polyunsaturated fatty acids (PUFAs), particularly of linoleic acid (LA, C18:2 n-6), enables animals to reach lower body temperatures (T_b_), lengthens torpor bout duration, and results in lower energy expenditure during hibernation. Conversely, dietary n-3 PUFA impacts negatively on hibernation performance. PUFA in surrounding phospholipids (PLs) presumably modulate the temperature-dependent activity of the sarcoplasmic reticulum (SR) Ca^2+^ ATPase 2 (SERCA2) and thus determine the threshold T_b_ still allowing proper heart function during torpor. We tested the effect of diets enriched with 10% of either corn oil (“CO,” high n-6 PUFA, e.g., LA) or menhaden oil [“MO,” long-chain n-3 PUFA, e.g., docosahexaenoic acid (DHA)] on hibernation performance and SERCA2 activity levels during torpor in garden dormice, an insectivorous, fat-storing hibernator. Prior to hibernation, individuals fed the MO diet showed an almost nine-times higher DHA levels and 30% lower LA proportions in white adipose tissue (WAT), reflecting the fatty acid composition of SR membranes, compared to CO-diet fed animals. When fed the MO diet, dormice significantly delayed their mean onset of hibernation by almost 4 days (range: 0–12 days), compared with CO-diet fed animals. Hibernation onset correlated positively with WAT-DHA levels and negatively with WAT-LA proportions prior to hibernation. Subsequently, hibernating patterns were similar between the two dietary groups, despite a significant difference in WAT-LA but not in WAT-DHA levels in mid-hibernation. SR-PL fatty acid composition and SERCA2 activity were identical in torpid individuals from the two dietary groups in mid-hibernation. In line with our previous findings on Syrian hamsters, a granivorous, food-storing hibernator, SERCA2 activity correlated positively with LA and negatively with DHA levels of SR-PL in torpid dormice, although SERCA2 activity was about three-times higher in garden dormice than in Syrian hamsters at similar PL-DHA proportions. Similarly, minimal T_b_ during torpor decreased as SERCA2 activity increased. We conclude that: (1) fatty acid composition of SR membranes modulates cardiac SERCA2 activity, hence determining the minimum T_b_ tolerated by hibernators, and (2) high DHA levels prevent hibernators from entering into torpor, but the critical levels differ substantially between species.

## Introduction

Dietary lipids strongly influence patterns of daily torpor and hibernation, i.e., states of reduced metabolic rate and hence body temperature (T_b_). Heterothermic mammals fed diets containing plant oils that are rich in polyunsaturated fatty acids (PUFAs) show a higher propensity of torpor use, lengthen their torpor bout duration, lower their minimum T_b_ and hence increase their energy savings (Geiser and Kenagy, 1987, 1993; Frank, 1992; Florant et al., 1993; Thorp et al., 1994; Bruns et al., 2000). However, we now know that there is no general effect of PUFA but specific, even conflicting effects of n-3 and n-6 PUFA (Ruf and Arnold, 2008; Giroud et al., 2013; Arnold et al., 2015).

In most of the experimental studies testing the effects of fatty acids on torpor and hibernation, it seems that the main PUFA added to the diet was linoleic acid (LA, C18:2), one of the main fatty acids of the n-6 family. Conversely, several studies showed adverse effects of n-3 PUFA on torpor and hibernation. To our knowledge, two studies (Hill and Florant, 2000; Frank et al., 2004) have tested the effects of a diet specifically enriched in α-linolenic acid (18:3 n-3) on hibernation, but only one (Hill and Florant, 2000) assessed the tissue fatty acid composition. The authors found that an n-3 PUFA enriched diet, leading to a high content in adipose tissues, strongly reduced the propensity to hibernate in yellow-bellied marmots (Hill and Florant, 2000). Despite of these clear antagonistic effects of n-6 and n-3 PUFA in modulating torpor and hibernation expression, the specific molecular mechanisms by which PUFA affect torpor are largely unknown although it is certainly not simply a reflection of lipid fluidity being higher due to incorporation of PUFA into phospholipids (PL) (Arnold et al., 2015).

A potential mechanistic role of PL n-6 PUFA in modulating torpor could be linked to the maintenance of the cardiac function at low T_b_ (Ruf and Arnold, 2008; Arnold et al., 2015). Indeed, the heart is a key organ that has to maintain circulation by regular contractions to guarantee sufficient perfusion of the organism. Whereas non-hibernators at T_b_ below 30°C experience severe arrhythmias and ventricular fibrillation leading to cardiac arrest, hibernating mammals remain in sinus rhythm even if T_b_ approaches 0°C (Johansson, 1996). This unique ability of the hibernator’s heart is due to the maintenance of sufficiently fast calcium removal into the sarcoplasmic reticulum (SR) after contraction, despite low T_b_ (see Wang et al., 2002; Andrews, 2007 for reviews). This process is under the control of the SR-calcium ATPase (SERCA2 in the heart), a key enzyme, for which gene expression and protein concentrations increase in preparation to hibernation (Yatani et al., 2004). Supporting evidence for a mechanistic role of PL-PUFA in maintaining the cardiac function at low T_b_ arises from a recent study in hibernating Syrian hamsters (*Mesocricetus auratus*), showing specific roles of certain PL n-6 and n-3 PUFA in regulating SERCA2. Cardiac SERCA activity was positively associated with the amount of LA and negatively with the amount of docosahexaenoic acid (DHA, 22:3 n-3) in cardiac SR PL of torpid hamsters (Giroud et al., 2013). Moreover, very high amounts of DHA in the SR PL were found in normothermic summer individuals, and animals that failed to hibernate in winter. Furthermore, SERCA2 activities altered by SR PL composition were negatively associated with the minimum T_b_ reached by Syrian hamsters during hibernation (Giroud et al., 2013). Hence, it appears that the PL fatty acid composition regulates cardiac SERCA activity during hibernation. These results were found, however, in animals fed identical rodent chows, and in a food storing hibernator, i.e., animals that continuously eat during the winter period. Further, these findings corresponded then to associations between SR composition, SERCA2 activity and T_b_, which ask for more experimental follow-up studies.

In the present study, we tested in the garden dormouse, a fat-storing hibernator, whether dietary supply during summer of n-6 PUFA (particularly LA) and long-chain n-3 PUFA (such as DHA) would shape PL-composition of tissues during winter and thus influence hibernation patterns and SERCA2 activity during deep torpor. We hypothesized that dormice fed a high n-3 PUFA (DHA) diet would: (i) either fully avoid torpor use or spend less time torpid and more time euthermic during the hibernation season, as previously observed in marmots (Hill and Florant, 2000), (ii) would show lower cardiac SERCA activity, and hence (iii) would hibernate at higher T_b_ during winter compared to animals fed a high n-6 PUFA (LA) diet.

## Materials and Methods

### Animals

Twenty-four garden dormice (*Eliomys quercinus*, 10 males, 14 females), 1–2 years old, from a breeding colony kept at the Research Institute of Wildlife Ecology (Vienna, Austria), were used in these experiments. Animals in our breeding colony were fed a standard diet consisting of dry hamster chows (Altromin 7024 Spezialfutter, GmbH & Co. KG, Lage, Germany), cat pellets (Topix, Saturn Petcare GmbH, Bremen, Germany) and sunflower seeds (prior to hibernation). During their pre-hibernation fattening, animals were housed in pairs in cages (60 × 40 × 40 cm), each equipped with two nests, bedding and nesting material. Animals were kept in a room under natural photoperiod, at a constant ambient temperature (T_a_) of 20°C, with *ad libitum* access to food and water. During the subsequent hibernation period (October 2011 to February 2012), dormice were housed individually in standard laboratory cages (36 × 20 × 14 cm), each provided with a customized nest and bedding material. Animals were housed in a room with windows but without artificial illumination and heating, i.e., exposed to natural fluctuations of photoperiod and T_a_, with access to water but not to food. T_a_ was gradually decreasing from 20 to 10°C during October and November, then down to 5.2 ± 1.9°C [-2.5 to +9.5°C CI] by December until February, i.e., the time of termination of experiments.

### Ethics Statement

All procedures were approved by the institutional ethics committee and the national Austrian authority according to §26 of Law for Animal Experiments, Tierversuchsgesetz 2013 – TGV 2013 (BMF – 68.205/0176-II/3b/2011).

### Diets

The diet formulae are summarized in **Table 1**. During the fattening phase, dormice were fed with specific diets, each differing in its lipid composition. These diets were made by adding a 10 wt% of refined corn oil as the source of n-6 fatty acids (mainly LA) or menhaden fish oil as the source of long-chain n-3 fatty acids (including DHA) to a pelleted purified ferret diet. We chose this pellet type to meet the nutritional demands of the largely insectivorous garden dormouse (Gil-Delgado et al., 2010; Kuipers et al., 2012), particularly its need for essential amino acids. We also chose menhaden fish oil as a source of n-3 fatty acids since it was used in previous studies reporting inhibitory effects of long chain n-3 fatty acids, notably DHA, on SERCA activity levels in rainbow trout skeletal muscle as well as in rat and mouse hearts (Swanson et al., 1989; Taffet et al., 1993; Ushio et al., 1997). All purified diets were supplemented with 2 wt% safflower oil to provide essential fatty acids. All dietary components were obtained from MP biomedical (Illkirch-Graffenstaden, France)^1^. This led to high amounts of DHA and LA in menhaden oil-enriched (“MO”) and corn oil-enriched diets (“CO”), respectively. The fatty acid compositions of the diets are summarized in **Table 2**. Pellets were kept in sealed bags filled with nitrogen at -80°C to minimize peroxidation until use. Fresh pellets were fed to the animals every 2 days, and uneaten food was discarded. During their fattening phase, dormice were offered both the standard diet used in our colony and one of the two specific diets at the start, and then after few days were fed exclusively on their respective specific diet, i.e., MO or CO diet. Due to the variability among the animals to habituate to their specific diet, each group of dormice consumed the experimental diet for at least 14 days, which has been shown by previous studies on small rodents to be sufficient to ensure maximum changes in the fatty acid composition of membrane PL (Swanson and Kinsella, 1986; Swanson et al., 1987).

**Table 1:** Composition of corn oil- (“CO”) and menhaden oil- (“MO”) enriched diets (expressed as % dry mass).

Ingredient	CO	MO
Casein, vitamin free	32.03	32.03
DL-Methionine	0.26	0.26
L-Arginine	0.20	0.20
Glucose	20.31	20.31
Cellulose	2.64	2.64
Soybean oil	25.00	25.00
Calcium phosphate	3.86	3.86
Choline bitartrate	0.18	0.18
Mineral mixture	3.52	4.00
Safflower oil	2.00	2.00
Corn oil	10.00	0.00
Menhaden oil	0.00	10.00

*Energy values were of 18.8 and 19.0 kJ.g^-1^ for CO and MO diets, respectively.*

**Table 2:** Fatty acid composition of corn oil- (“CO”) and menhaden oil- (“MO”) enriched diets as fed to garden dormice for at least 3 weeks during their pre-hibernation fattening phase.

Fatty acid	CO	MO
C14:0	0.28	3.23
C15:0	0.03	0.25
C16:0	12.01	14.69
C16:1 (n-7)	0.18	4.26
C17:0	0.08	0.80
C18:0	1.65	2.38
C18:1 (n-9)	26.93	21.17
C18:2 (n-6)	57.37	39.14
C18:3 (n-3)	0.97	1.11
C20:4 (n-6)	0.01	0.51
C20:5 (n-3)	0.12	6.75
C22:5 (n-3)	0.04	1.22
C22:6 (n-3)	0.35	4.49
PUFA	58.85	53.22
MUFA	27.10	25.43
SFA	14.05	21.35
Σn-6	57.38	39.65
Σn-3	1.47	13.56
n-6/n-3	38.99	2.92

*Fatty acid proportions are expressed as % of total fatty acids. Values are means from two analyses per diet. PUFAs, polyunsaturated fatty acids; MUFAs, monounsaturated fatty acids; SFAs, saturated fatty acids; Σn-6, the sum of n-6 PUFA; Σn-3, the sum of n-3 PUFA; and n-6/n-3, the ratio between the sum of n-6 PUFA and the sum of n-3 PUFA.*

### Protocol Overview

During the pre-hibernation fattening phase, dormice were fed one of the two specific diets for 17–21 days in September 2011 (start: 5.9; end: 21–25.9). Weekly body mass and food intake were assessed during this period. Food intake was measured by weighing the amount of food provided to each dormouse and the food left in the cages (including spillage) to the nearest gram (PM34 Delta Range, Mettler Toledo, Greifensee, Switzerland). The amount of consumed energy was computed using the energy values of the respective diet (**Table 1**). The diet energy values were calculated from the energy contents of the pelleted purified ferret diet provided by MP biomedical, of safflower oil and of either corn oil or menhaden fish oil. Gross energy intake was calculated from the amount of food consumed in grams during the period of dietary treatment multiplied by its corresponding energy content. After the pre-hibernation fattening phase all 24 individuals were implanted with small temperature transmitters (see below), and subcutaneous WAT was sampled, except for one animal from the CO group and one from the MO group, in order to quantify the effect of dietary treatments on WAT fatty acid composition. After surgery, individuals were allowed a recovery phase of 2 weeks, during which they were fed their respective specific diet. Unfortunately, one dormouse from the CO group died during that period, reducing the number of experimental animals to 23 (10 males, 13 females). In early-October, hibernation was induced by removing food. Hibernating patterns were monitored during the next 4 months (see below for details) until animals were sacrificed in mid-hibernation (February 2012). All animals were sacrificed within a 2 weeks period by decapitation after ∼4 days in deep torpor (T_b_∼4°C). Hearts and peritoneal WAT were quickly sampled. Hearts were immediately processed for isolation of cardiac SR (see below for further details). WAT were flash-frozen in liquid nitrogen and stored at -80°C for subsequent analyses.

### Surgical Implantations of Transmitters and T_b_ Measurements

Transmitters (model: TA-10TA-F20, 1.75 cc, 3.8 g, accuracy: 0.15°C; Data Sciences International, Saint Paul, MN, United States) were calibrated prior to implantation between 0 and 40°C in a temperature-controlled water bath. Transmitters were surgically implanted under anesthesia induced by subcutaneous injection of 50 mg kg^-1^ ketamine (Ketamidor^®^ 10%, Richter Pharma, Wels, Austria) and 5 mg kg^-1^ xylazine (Rompun^®^ 2%, Bayer, Leverkusen, Germany). Animals were then maintained with 1.5% isoflurane in an oxygen stream via facemask. For post-operative analgesia 5 mg kg^-1^ ketoprofen (Romefen 10% Merial S.A.S., Toulouse, France) was administered subcutaneously. The operation field was prepared according to standard surgical procedures and covered by sterile surgical drapes. Animals were placed in dorsal recumbency and the abdominal cavity was opened through a 1 cm incision in the *linea alba* to introduce the temperature transmitter within the abdomen and to collect small amounts (10–30 mg) of subcutaneous WAT. WAT-samples were flash-frozen in liquid nitrogen and stored at -80°C until subsequent analysis for fatty acid composition. Peritoneum and abdominal muscles were sutured using synthetic absorbable surgical suture material USP 3/0 (Surgicryl PGA, SMI AG, Hünningen, Belgium) using a single-button suture technique. In addition synthetic absorbable surgical suture material USP 4/0 (Surgicryl PGA, SMI AG, Hünningen, Belgium) was used to suture the skin with an intra-cutaneous suture technique. During the entire procedure, vital parameters [respiration rate, peripheral hemoglobin oxygen saturation as measured by pulse oximetry (SpO_2_), and heart rate] were monitored. After surgery, all animals recovered for a period of 2 weeks before starting temperature recordings.

A receiver board (RPC-1; Data Sciences International, Saint Paul, MN, United States) was positioned under each individual cage to collect the radio frequency signals from transmitters. T_b_ was recorded for 10 s every 5 min. Data were analyzed using the Dataquest software (LabPro Data Sciences). Several parameters were derived from the temperature recordings. We determined the onset of hibernation as the time between the food removal and entrance into the first torpor bout with a T_b_ threshold of 25°C and lasting at least for 24 h. We also computed the number of arousals, mean and total arousal and torpor durations (with a T_b_ threshold of 25°C), and minimal T_b_ during torpor.

### Isolation of Cardiac SR

Cardiac homogenates were prepared from approximately half of each dormouse heart (∼130 mg), as described previously (Giroud et al., 2013). Hearts were washed and minced in ice-cold isotonic saline (0.9% NaCl). A protease inhibitor cocktail (Sigma P8340) was added to the suspensions. Tissues were then homogenized in 2 ml of a buffer containing 30 mM histidine (pH 6.9), 300 mM sucrose, 600 mM KCl, 0.5 mM DTT, 10 mM EDTA, 50 mM Na_2_HPO_4_ and 1 mM PMSF, by 10 strokes with a motor-driven Teflon/glass homogenizer (tube volume, 5 ml, Wheaton, IL, United States). Homogenates were then centrifuged at 24,600 *g* for 15 min to remove mitochondria, cell debris and most of the membranes, including sarcolemma, but not SR (Meissner et al., 1973). Homogenates therefore contained SERCA2 protein embedded in membrane vesicles, and SERCA2 represents the major fraction (∼60–80%) of the total membrane protein of the longitudinal SR (Inesi, 1972; Hasselbach, 1974). Supernatants were stored in 200 μl-aliquots at -80°C for subsequent SERCA2 activity measurements. All steps were performed at 4°C within 1 h to minimize enzyme denaturation.

### SERCA2 Activity Measurements

ATPase activities were measured by a standard coupled enzyme assay, in which the rate of ATP hydrolysis was calculated from spectrophotometric recording (Perkin-Elmer 550, Germany) of NADH oxidation at 340 nm (𝜀 = 6.22 mM^-1^ cm^-1^). The assay was performed according to the method previously described by Simonides and van Hardeveld (1990), using a sample volume optimized for the garden dormouse’s heart. SERCA2 activity was determined by adding 100 nM thapsigargin (TG), a specific inhibitor of SERCA (Lytton et al., 1991), during the assay. Therefore, the difference between ATPase activities recorded before and after TG addition can be attributed to SERCA2. The standard reaction mixture contained 50 mM imidazole (pH 6.9), 100 mM KCl, 10 mM MgCl_2_, 10 mM NaN_3_, 0.5 mM DTT, 10 mM PEP, 5 mM ATP, 10 μM CaCl_2_, 5.3 unit.ml^-1^ pyruvate kinase, 17.5 unit.ml^-1^ lactate dehydrogenase, 300 μM NADH and 2 μM calcium ionophore (A23187) in a final volume of 1 ml. The reaction was started by addition of the sample (0.1–0.2 μg μl^-1^ final volume). Assays were performed at 37°C. Protein concentrations were determined with the Bradford method (Bradford, 1976). Data presented are means from three replicate measurements of SERCA2 activities, and are expressed as μmol ATP hydrolyzed per mg total protein and minute.

### Lipid Analyses

Total lipids were extracted from both WAT and isolated SR membranes following the procedure of Folch et al. (1957). Since triglyceride fatty acids represent >95% of total lipids in rodent WAT (Florant et al., 1990), triglycerides and PLs in WAT were not separated prior to analysis. Samples were trans-esterified with a one-step method (Lepage and Roy, 1986; Eder, 1995). As for SR membrane lipids, PLs were isolated using thin-layer chromatography (Christie, 2005), and further saponified and converted into fatty acid methyl esters (FAME) as reported previously (Eder, 1995). FAME were identified by gas-liquid chromatography using a Perkin-Elmer FID AutoSystem XL autosampler chromatograph equipped with a 30 m × 0.25 mm × 0.25 μm HP INNOWax capillary column, using the following parameters: injector 240°C, column 130–180°C at 4°C/min, 180–200°C at 3°C/min, 200–240°C at 15°C/min, 240°C for 8 min. The relative fatty acid composition was quantified using external FAME standards (Supelco) run after every 20 samples and Turbochrom 6.3 software (Perkin-Elmer). Concentrations of single fatty acids were calculated as mass % of total identified peaks of 13 fatty acids of chain length 14–22. Due to mistakes in the lipid extraction phase, samples from two individuals (one from each dietary group) were excluded from the results of the fatty acid composition for SR PL.

### Statistical Analysis

Data analyses were carried out using R 3.1.1 (R Core Team, 2014). The normality of residuals from statistical models was assessed by inspecting quantile–quantile-plots and histograms. If necessary, response variables were Box–Cox transformed to achieve normality. We employed linear models to compare body mass of dormice from CO and MO groups prior to their diet treatment, and to determine effects of dietary treatment (CO vs. MO) and sex on body mass prior to hibernation and energy intake of animals during the period of the feeding treatment. We used linear mixed-effects models (R package “nlme”) (Pinheiro et al., 2014) with animal ID as random factor to determine effects of dietary treatment (CO vs. MO) and time (pre-hibernation vs. mid-hibernation) on WAT fatty acid composition. Tukey-like *post hoc* multiple comparison tests (R package “multcomp”) (Hothorn et al., 2008) were applied to test for differences between groups (CO vs. MO) and periods (pre-hibernation vs. mi-hibernation). General linear models with Tukey-like *post hoc* multiple comparison tests were used to assess differences in SR PL fatty acid composition between the two dietary groups (MO vs. CO). All *p*-values from linear mixed-effects models and general linear models were adjusted for multi-comparisons between fatty acid proportions using False Discovery Rate (Benjamini and Hochberg, 1995). We used analysis of variance to test effects of diets on variables derived from the hibernation patterns, except for the number of arousals for which we used a generalized linear model with a Poisson distribution. This GLM showed no signs of over-dispersion. Body mass was entered as a random factor in the model for hibernation onset. Initial inspection of the data gave no evidence for a sex effect on any of the response variables derived from the hibernating patterns, including hibernation onset. Since animals were sacrificed within a period of 2 weeks, individual experimental hibernation duration was also included as a random factor in the model for number of arousals as well as total arousal and torpor durations. We also used an analysis of variance to test for effect of diets on SERCA activity levels at mid-hibernation. For regression analyses of variables derived from hibernation patterns vs. SERCA activity levels, we applied ranged major axis (RMA) models (R package “lmodel2”) (Legendre and Legendre, 1998) to account for measurement errors in both the dependent and predictor variable. All reported values are means ± standard deviation.

## Results

### Body Mass and Energy Intake During the Pre-hibernation Fattening Phase

At the start of the feeding treatments, dormice from the two dietary groups had almost identical mean body mass, although males were heavier than females (**Table 3** and **Supplementary Table S1**). During pre-hibernation fattening, daily energy intake did not differ between dietary groups, but was significantly higher in females compared to males (**Table 3** and **Supplementary Table S1**). After the feeding treatments, both dietary groups and sexes showed similar body mass (**Table 3** and **Supplementary Table S1**).

**Table 3:** Body mass (means ± standard deviation) at the start of the diet treatment (“Pre-diet”) and prior to hibernation (“Pre-hibernation”), and energy intake (means ± standard deviation) during the diet treatment of male (“M”) and female (“F”) garden dormice fed diets enriched with either n-6 fatty acids (“CO”) or n-3 fatty acids (“MO”).

Dietary groups	Body mass (g)				Energy intake (kJ.day^-1^)	
	Pre-diet		Pre-hibernation		M	F
	M	F	M	F		
CO	150.6 ± 4.2	118.3 ± 27.2	163.6 ± 9.5	144.4 ± 22.7	189.0 ± 38.6	209.9 ± 16.0
MO	139.6 ± 15.0	119.1 ± 13.0	142.6 ± 4.7	150.6 ± 14.5	151.8 ± 52.9	202.5 ± 24.5

### Lipid Profiles of WAT Prior to Hibernation and at Mid-Hibernation

After 3 weeks of a specific dietary treatment (CO vs. MO) prior to hibernation, the fatty acid composition of WAT significantly differed according to the dietary lipids being consumed (**Table 4** and **Supplementary Table S2**). The dormice fed the MO diet, had an almost nine-times higher level of DHA (C22:6 n-3), a 15-times higher level of eicosapentaenoic acid (EPA, C20:5 n-3), a five-times higher level of docosapentaenoic acid (DPA, C22:5 n-3) and a 30% lower proportion of LA (C18:2 n-6) in their WAT, compared with those fed the CO diet (**Table 4** and **Supplementary Table S2**). These differences summed up to a 30% lower level of n-6 PUFA and a 2.3-times higher level of n-3 PUFA in animals fed the MO diet compared to those fed the CO diet. We also found that proportions of myristic acid (C14:0) and palmitic acid (C16:0) significantly differed between dietary groups. The animals fed a MO diet had slightly higher levels of these two saturated fatty acids (SFAs), leading to a moderate but significant 15% larger proportion of SFA in their WAT. Levels of palmitoleic acid (C16:1 n-7) were also slightly higher in MO diet-fed dormice compared to animals fed a CO diet.

**Table 4:** Fatty acid proportions (% of total fatty acids), prior to and at mid-hibernation of white adipose tissue total lipids (means ± standard deviation) and ratios of certain fatty acid proportions of garden dormice fed diets enriched with either n-6 fatty acids (“CO”) or n-3 fatty acids (“MO”).

Fatty acid	Pre-hibernation		Mid-hibernation	
	CO	MO	CO	MO
C14:0	1.40 ± 0.19^a^	1.99 ± 0.26^b^	1.14 ± 0.12^c^	2.08 ± 0.40^b^
C15:0	0.13 ± 0.06^a^	0.16 ± 0.09^a^	0.01 ± 0.01^b^	0.01 ± 0.01^b^
C16:0	13.03 ± 1.00^a^	15.07 ± 1.66^b^	9.00 ± 0.74^c^	11.12 ± 1.54^d^
C16:1 n-7	4.83 ± 0.64^a^	6.23 ± 0.83^b^	3.20 ± 0.40^c^	4.72 ± 0.83^a^
C17:0	0.15 ± 0.07^ac^	0.24 ± 0.10^a^	0.00 ± 0.00^b^	0.06 ± 0.11^bc^
C18:0	2.43 ± 0.31^ab^	2.88 ± 0.56^a^	2.04 ± 0.28^b^	2.49 ± 0.46^ab^
C18:1 n-9	44.82 ± 4.89^a^	45.53 ± 4.75^a^	43.66 ± 3.77^a^	46.54 ± 5.35^a^
C18:2 n-6	30.00 ± 4.56^a^	21.06 ± 2.47^b^	38.51 ± 3.87^c^	30.28 ± 3.35^a^
C18:3 n-3	2.34 ± 0.43^a^	2.35 ± 0.44^a^	2.23 ± 0.48^a^	2.21 ± 0.53^a^
C20:4 n-6	0.45 ± 0.10^a^	0.59 ± 0.23^a^	0.21 ± 0.17^b^	0.15 ± 0.20^b^
C20:5 n-3	0.12 ± 0.09^a^	1.80 ± 0.55^b^	0.00 ± 0.00^a^	0.17 ± 0.20^a^
C22:5 n-3	0.11 ± 0.08^a^	0.54 ± 0.16^b^	0.00 ± 0.00^c^	0.06 ± 0.11^ac^
C22:6 n-3	0.18 ± 0.08^a^	1.58 ± 0.52^b^	0.00 ± 0.00^a^	0.11 ± 0.17^a^
PUFA	33.21 ± 5.06^a^	27.91 ± 3.82^b^	40.95 ± 4.36^c^	32.98 ± 3.86^a^
MUFA	49.65 ± 5.25^a^	51.76 ± 5.24^ab^	46.86 ± 4.08^b^	51.26 ± 5.28^ab^
SFA	17.14 ± 1.26^a^	20.33 ± 2.34^c^	12.18 ± 0.95^c^	15.75 ± 2.18^a^
Σn-6	30.45 ± 4.55^a^	21.65 ± 2.40^b^	38.72 ± 3.92^c^	30.43 ± 3.35^a^
Σn-3	2.76 ± 0.58^a^	6.26 ± 1.62^b^	2.23 ± 0.48^a^	2.55 ± 0.86^a^
n-6/n-3	11.23 ± 1.32^a^	3.66 ± 0.92^b^	17.76 ± 2.10^c^	12.92 ± 3.52^a^

*PUFAs, polyunsaturated fatty acids; MUFAs, to monounsaturated fatty acids; SFAs, saturated fatty acids; Σn-6, the sum of n-6 PUFA; Σn-3, the sum of n-3 PUFA; n-6/n-3, the ratio between the sum of n-6 PUFA and the sum of n-3 PUFA. Groups and periods differing significantly with *p* < 0.05 (Tukey-like *post hoc* comparisons) are denoted by different superscripts.*

At mid-hibernation, differences in fatty acid composition of WAT between dietary groups were diminished compared to prior to hibernation (**Table 4** and **Supplementary Table S2**). DHA proportions in WAT were significantly reduced in MO-fed dormice during hibernation and did not differ anymore between dietary groups at mid-hibernation (**Table 4** and **Supplementary Table S2**). EPA and DPA proportions as well as the sum of n-3 PUFA also decreased in MO-fed dormice during hibernation, and were similar between dietary groups at mid-hibernation. Simultaneously, levels of LA increased dramatically in both groups from onset of hibernation to mid-hibernation, and were still 20% lower in WAT of MO diet-fed dormice compared to CO diet-fed animals at mid-hibernation (**Table 4** and **Supplementary Table S2**). Hence, individuals fed a MO diet had a significant 20% lower level of n-6 PUFA, but a similar proportion of n-3 PUFA, compared to animals fed a CO diet. Further, proportions of myristic acid (C14:0) and palmitic acid (C16:0) significantly differed between dietary groups, leading to slightly 20% higher levels of SFA in dormice fed a MO diet vs. fed a CO diet, at mid-hibernation. Levels of palmitoleic acid (C16:1 n-7) were also slightly higher in MO diet-fed dormice compared to animals fed a CO diet.

### Hibernating Patterns

**FIGURE 1:**
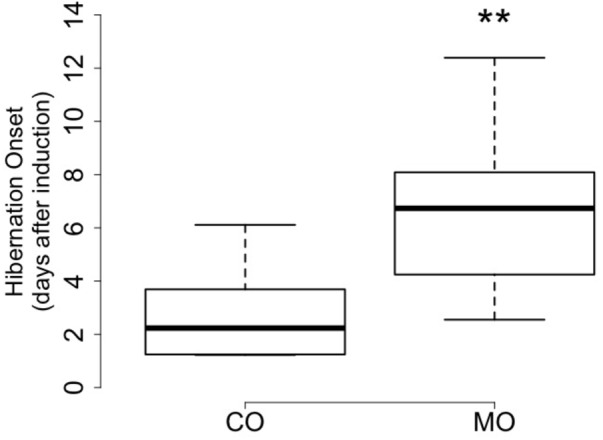
Number of days until onset of hibernation after removal of food and water in October of by garden dormice fed the n-6 PUFA-enriched (“CO”) or n-3 PUFA-enriched (“MO”) diet (boxplots, ^∗∗^*p* < 0.01).

At the onset of hibernation, body mass did not differ between dormice fed a MO diet and individuals fed a CO diet (148.1 ± 19.3 vs. 161.4 ± 19.4 g, *F* = 2.27, *p* = 0.15). However, the onset of hibernation significantly differed between the MO and CO groups (**Figure 1**). Dormice fed the MO diet significantly delayed mean hibernation onset by almost 4 days (range: 0–12 days), compared to those fed the CO diet (**Table 5**). Thereafter, no differences in hibernation patterns were detected between animals of the two dietary groups. The number of arousals, mean and total arousal durations, mean and total torpor durations, as well as the mean minimal T_b_ did not differ between dietary groups (**Table 5**).

**Table 5:** Variables of hibernating patterns of garden dormice fed diets enriched with either n-6 fatty acids (“CO”) or n-3 fatty acids (“MO”).

Variables	CO	MO	ANOVA	
			*t*-Value	*p*-Value
Hibernation onset (days)	2.7 ± 1.9	6.5 ± 2.8	3.39	*<0.01*
Number of arousals	14.0 ± 2.1	13.7 ± 2.5	–0.31	0.76
Mean arousal duration (h)	9.3 ± 1.6	8.6 ± 1.2	–1.14	0.27
Total arousal duration (h)	118.9 ± 16.1	108.4 ± 26.0	–1.02	0.32
Mean torpor duration (h)	200.5 ± 24.3	200.3 ± 35.5	–0.02	0.99
Total torpor duration (h)	2766.4 ± 143.0	2659.1 ± 148.2	–1.61	0.13
Minimal body temperature (°C)	–0.42 ± 0.95	–0.45 ± 0.56	–0.09	0.93

*Body mass was accounted as a random factor in the model for hibernation onset. Winter duration was also included as a random factor in the model for number of arousals and, total arousal and torpor durations. Significant *p*-values are shown in italic.*

### Hibernation Patterns and WAT Lipid Composition Prior to Hibernation

**FIGURE 2:**
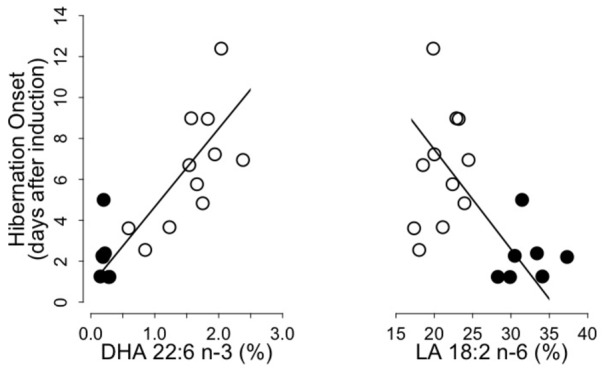
Onset of hibernation as a function of Docosahexaenoic acid (“DHA 22:6 n-3,” left panel) and Linoleic acid (“LA 18:2 n-6,” right panel) levels in WAT total lipids of garden dormice fed either a n-6 PUFA-enriched (black dots) or a n-3 PUFA-enriched diet (open dots). Regression statistics from linear ranged major axis analysis: (DHA) intercept = 0.83, slope = 3.83, adjusted *R*^2^ = 0.65, *p* < 0.001; (LA) intercept = 17.20, slope = –0.49, adjusted *R*^2^ = 0.25, *p* < 0.01.

We found significant positive and negative relationships between the hibernation onset and levels of DHA and LA, respectively, of WAT total lipids prior to hibernation (**Figure 2**). Hibernation onset was also positively and negatively associated with the sums of n-3 PUFA and n-6 PUFA, respectively, as well as negatively with the n-6/n-3 PUFA ratio (n-3 PUFA: intercept = -2.68, slope = 1.50, adjusted *R*^2^ = 0.64, *p* = 0.001; n-6 PUFA: intercept = 17.82, slope = -0.50, adjusted *R*^2^ = 0.24, *p* = 0.01; n6/n3 PUFA ratio: intercept = 9.80, slope = -0.77, adjusted *R*^2^ = 0.51, *p* = 0.001). Further, hibernation onset increased with pre-hibernation levels of both EPA and DPA of WAT total lipids (EPA: intercept = 1.09, slope = 3.25, adjusted *R*^2^ = 0.64, *p* = 0.001; DPA: intercept = -0.08, slope = 12.98, adjusted *R*^2^ = 0.61, *p* = 0.001). We did not find any further correlations between other variables of the hibernating patterns and the fatty acids composition of WAT lipids.

### SR Phospholipid Composition and Cardiac SERCA Activity in Deep Torpor

**FIGURE 3:**
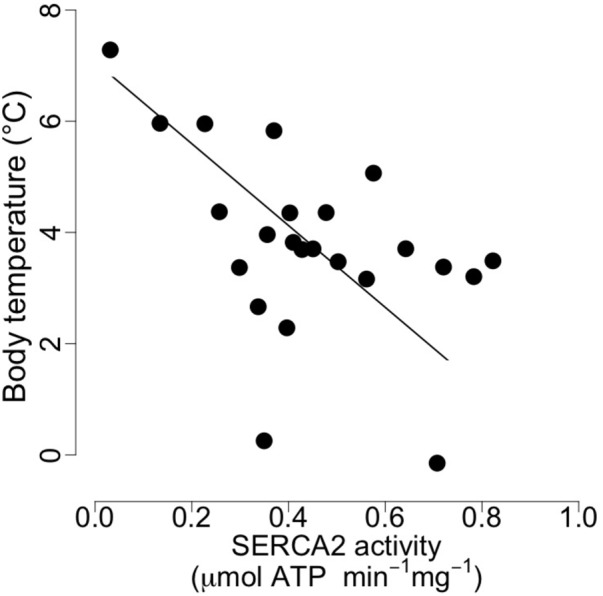
Body temperature as a function of cardiac sarcoplasmic reticulum (SR) Calcium ATPase 2a (“SERCA”) activity in garden dormice during deep torpor (mid-hibernation). Regression statistics from linear ranged major axis analysis: intercept = 7.07, slope = –7.36, adjusted *R*^2^ = 0.21, *p* = 0.01.

At mid-hibernation, no significant differences in the fatty acid composition of SR PL were found between the two dietary groups (**Table 6**). Further, levels of SERCA2 activity (MO vs. CO: 0.50 ± 0.23 vs. 0.39 ± 0.16 μmol. ATP min^-1^ mg^-1^, *F* = 1.79, *p* = 0.19), and minimal T_b_ during torpor (MO vs. CO: 3.83 ± 1.80 vs. 3.75 ± 1.59°C, *F* = 0.01, *p* = 0.90) did not differ significantly. However, SERCA2 activity was negatively associated with minimal T_b_ of the animals during torpor (**Figure 3**).

**Table 6:** Fatty acid proportions (% of total fatty acids), at mid-hibernation, of cardiac sarcoplasmic reticulum phospholipids (means ± standard deviation) and ratios of certain fatty acid proportions of garden dormice fed diets enriched with either n-6 fatty acids (“CO”) or n-3 fatty acids (“MO”).

Fatty acid	CO	MO	ANOVA	
			F-Statistic	*p*-Value
C14:0	0.48 ± 0.23	0.47 ± 0.32	0.002	0.96
C15:0	0.23 ± 0.20	0.24 ± 0.20	0.02	0.95
C16:0	11.29 ± 1.54	13.00 ± 1.88	5.10	0.14
C16:1 (n-7)	0.45 ± 0.15	0.57 ± 0.16	2.80	0.21
C17:0	0.36 ± 0.22	0.44 ± 0.31	0.56	0.56
C18:0	14.83 ± 2.54	17.96 ± 5.04	3.13	0.21
C18:1 (n-9)	8.58 ± 2.31	10.45 ± 2.79	2.77	0.21
C18:2 (n-6)	25.04 ± 4.91	23.75 ± 2.99	0.54	0.56
C18:3 (n-3)	0.76 ± 0.38	0.73 ± 0.54	0.02	0.95
C20:4 (n-6)	8.27 ± 2.52	6.66 ± 1.81	2.89	0.21
C20:5 (n-3)	0.18 ± 0.31	1.63 ± 1.43	9.80	0.05
C22:5 (n-3)	1.12 ± 0.39	2.31 ± 1.01	12.17	0.05
C22:6 (n-3)	28.41 ± 13.16	21.78 ± 10.60	1.63	0.37
PUFA	63.78 ± 5.76	56.86 ± 6.52	6.57	0.11
MUFA	9.04 ± 2.23	11.02 ± 2.75	3.25	0.21
SFA	27.19 ± 3.59	32.12 ± 5.31	6.09	0.11
Σn-6	33.32 ± 7.34	30.41 ± 4.32	1.25	0.44
Σn-3	30.46 ± 12.96	26.45 ± 9.44	0.67	0.56
n-6/n-3	1.68 ± 1.65	1.29 ± 0.46	0.58	0.56
LA/DHA [C18:2/C22:6]	1.52 ± 1.68	1.32 ± 0.60	0.13	0.72

*PUFAs, polyunsaturated fatty acids; MUFAs, monounsaturated fatty acids; SFAs, saturated fatty acids, Σn-6, the sum of n-6 PUFA; Σn-3, the sum of n-3 PUFA; n-6/n-3, the ratio between the sum of n-6 PUFA and the sum of n-3 PUFA.*

**FIGURE 4:**
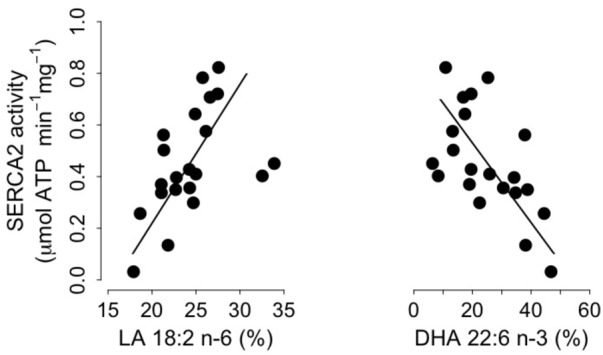
Cardiac Sarcoplasmic Reticulum (SR) Calcium ATPase 2a (“SERCA”) activity as a function of the proportions (% of total fatty acids) of Linoleic acid (“LA 18:2 n-6,” left panel) and Docosahexaenoic acid (“DHA 22:6 n-3,” right panel) in SR phospholipids. Regression statistics from linear ranged major axis analysis: (LA) intercept = –0.86, slope = 0.05, adjusted *R*^2^ = 0.20, *p* = 0.01; (DHA) intercept = 0.84, slope = –0.02, adjusted *R*^2^ = 0.33, *p* = 0.002.

We found significant positive and negative correlations between levels of SERCA2 activity and proportions of both LA and DHA, which are the main n-6 and n-3 PUFA respectively, in SR PL (**Figure 4**). Moreover, SERCA2 activity was also correlated with the sums of n-6 PUFA and n-3 PUFA in SR PL (n-6 PUFA: intercept = -0.73, slope = 0.04, adjusted *R*^2^ = 0.17, *p* = 0.02; n-3 PUFA: intercept = 0.95, slope = -0.02, adjusted *R*^2^ = 0.30, *p* = 0.002). SERCA2 activity increased, however, with the proportions of EPA and DPA, precursors of DHA, in SR PL of torpid dormice (EPA: intercept = 0.35, slope = 0.11, adjusted *R*^2^ = 0.14, *p* = 0.03; DPA: intercept = 0.08, slope = 0.22, adjusted *R*^2^ = 0.10, *p* = 0.04), but proportions of those fatty acids were only <3%. Also, no significant association existed between arachidonic acid (C20:4 n-6) and SERCA2 activity. However, we also found significant associations between SERCA2 activity levels and the proportion of oleic acid (C18:1 n-9) and the sum of monounsaturated fatty acids (MUFAs) (oleic acid: intercept = -0.21, slope = 0.07, adjusted *R*^2^ = 0.13, *p* = 0.03; MUFA: intercept = -0.23, slope = 0.07, adjusted *R*^2^ = 0.13, *p* = 0.03).

## Discussion

### High Levels of Docosahexaenoic Acid Delay the Onset of Hibernation

In this study, we found that dormice fed a MO diet, i.e., with high levels of DHA and low proportions of LA, delayed their onset of hibernation, compared to individuals fed a CO diet with high LA and low DHA contents (**Figures 1, 2** and **Table 5**). These results are in line with findings of Hill and Florant (2000) reporting inhibitory effects of diets enriched with linolenic acid (18:3 n-3), the precursor of DHA, on hibernation propensity in yellow-bellied marmots. Only three marmots entered hibernation on eight individuals tested, whereas five other animals stayed euthermic throughout winter and continued to feed. Similar findings were reported in a daily heterotherm, the gray mouse lemur (Vuarin et al., 2016). Dietary supplementation with n-3 PUFA (mainly EPA and DHA) during 9 weeks reduced torpor use in mouse lemurs. Whereas animals fed a standard diet progressively deepened their torpor bouts, mouse lemurs provided with a n-3 PUFA enriched-diet never entered into torpor but instead expressed only shallow reductions in T_b_ during the first 2 weeks of the experiment, then showed shallower and shorter torpor bouts during the following 7 weeks. Our results are also in agreement with previous findings in Syrian hamsters, a food-storing hibernator (Giroud et al., 2013). Indeed, a group of hamsters did not hibernate during the entire winter and showed very high levels of DHA in their cardiac SR membrane PL, similar to animals in summer, but unlike animals that did hibernate during winter. Interestingly, SERCA2 activity was also lower in these hamsters in comparison to that of hamsters entering torpor. It has been reported that n-3 PUFA suppress SERCA2 activity (Swanson et al., 1989; Taffet et al., 1993; Ushio et al., 1997), and high levels of n-3 PUFA, notably DHA, in the heart have been suggested to be detrimental for proper cardiac function at low T_b_ during torpor (Ruf and Arnold, 2008; Giroud et al., 2013). Taken together, these findings suggest that the delayed hibernation onset observed in dormice fed a MO diet was due to the specific inhibitory effect of DHA on SERCA2 activity, preventing animals to enter hibernation.

### Diet-Independent Remodeling During Hibernation

Unlike the Syrian hamster, the garden dormouse is a fat-storing hibernator. Depending on the location, dormice can hibernate for several months during winter and rely largely on body fat reserves during hibernation. In this study, garden dormice were hence fasted from the winter start, to induce the use of torpor and trigger hibernation. We found that proportions of n-3 PUFA and notably DHA in both WAT and SR PL at mid-hibernation were almost identical between the two dietary groups (**Tables 4, 6**), whereas marked group differences in WAT proportions of n-3 PUFA were observed prior to hibernation (**Table 4**). This suggests that MO diet-fed dormice may have had to reduce their DHA levels in their tissues and PL membranes before entering into torpor and starting hibernation. This finding contrasts with previous studies on marmots (Hill and Florant, 2000), gray mouse lemurs (Vuarin et al., 2016), and Syrian hamsters (Giroud et al., 2013), in which individuals with high tissue levels of n-3 PUFA did enter torpor or hibernate during the entire winter. Interestingly, the animals from the studies cited above had the possibility in their respective experimental setup to continuously feed, hence providing a source for the intake of n-3 PUFA. In contrast, garden dormice of this study were fasted from the winter start, constraining them to possibly reduce levels of DHA in their tissues and PL membranes and to start hibernating in order to survive the winter without food. Further, proportions of LA increased in both groups over winter hibernation (**Table 4**). Similar findings in alpine marmots also suggested that the drop in T_b_ in fall was paralleled by a massive incorporation of LA into heart and liver membranes (Arnold et al., 2011). Such changes would have been possible through transfer between tissues and/or specific oxidations of DHA and LA, as it has already been reported in fasted laboratory rats and food-restricted mouse lemurs (Raclot, 2003; Giroud et al., 2009). Potential molecular mechanisms could involve the glycerol-3-phosphate acyltransferase 1 (GPAT1), which is found in the outer mitochondrial membrane and catalyzes the common first step of triglyceride and PL synthesis. GPAT1 preferentially esterifies palmitate (C16:0) at the sn-1 position of the glycerol-3-phosphate. In the absence of GPAT1 activity, the C16:0 content is lower in PL, and accompanied by substantial enrichment of n-6 PUFA, namely arachidonic acid (C20:4 n-6), and a decrease of DHA at the sn-2 position. Since GPAT1 mRNA is known to decrease with fasting, it could well be a mechanism explaining the fast removal of DHA in MO diet-fed individuals during winter hibernation (see Arnold et al., 2015 for review).

Another reason for a fast removal of DHA prior to the entrance in hibernation, i.e., a state of hypometabolism, probably lies in the fact that n-3 PUFAs are known to have positive effects on metabolic pathways, i.e., those delivering ATP (Power and Newsholme, 1997). In particular, DHA in PL seems to be linked to increases of energy production. Indeed, DHA-enriched cells showed increased mitochondrial potential, i.e., the electrical gradient between the inner and outer mitochondrial membranes needed for ATP production, and produced five-times more cellular oxidants than did cells enriched with any other fatty acid (Watkins et al., 1998). Further, its has been suggested that DHA increases cell oxidant production by accumulating cardiolipin, one major PL found in the inner mitochondrial membrane (Hostetler, 1982), where its presence alters electron transport efficiency (Watkins et al., 1998). In particular, the F_0_F_1_ ATPase in the mitochondrial inner membrane appears to have high affinity for cardiolipin (Eble et al., 1990). Therefore the removal of DHA in MO-diet fed dormice might have been required for the animals before being able to enter a hypometabolic state during hibernation.

Taken together, these results suggest that dormice fed CO and MO diets prior to hibernation specifically remodeled their tissues and membranes during the subsequent winter. Individuals fed a MO diet drastically reduced levels of DHA in tissues and membranes, probably prior to hibernation. Further, animals increased proportions of LA, irrespectively of diets, in their tissues and membranes over winter. Such effects known as selective retention have been previously described in chick embryo, neonatal rats and hibernating marmots (Xia et al., 1993).

### Specific Roles of LA and DHA in Regulating SERCA2 Activity During Torpor

Apart from the delay of the hibernation onset, hibernating patterns did not significantly differ between dietary groups (**Table 5**), although dormice fed a CO diet had higher levels of LA in their WAT prior to hibernation and at mid-hibernation, compared to animals fed a MO diet (**Table 4**). In contrast to WAT, the fatty acid composition, notably heart SR PL levels of LA (∼23–25%) was similar between the two dietary groups (**Table 6**). Similar LA contents were reported in heart SR PL of marmots (∼26%, Arnold et al., 2011) immediately after immergence from hibernation, and in hibernating Syrian hamsters (∼24%, Giroud et al., 2013). This supports the view that the heart is a key organ to preserve during hibernation, and that SR PL fatty acid composition is tightly regulated to ensure proper cardiac function through the maintenance of cardiac SERCA activity at low T_b_. Indeed, we found significant relationships between SR PL contents of LA and SERCA2 activity (**Figure 4**), as well as between SERCA2 activity (measured at 37°C) and minimum T_b_ reached in torpor (**Figure 3**), as also reported in hibernating Syrian hamsters (Giroud et al., 2013).

In this study, we also found significant correlations between heart SR PL levels of DHA (and n-3 PUFA), SERCA2 activities and T_b_ in torpid garden dormice at mid-hibernation (**Figures 3, 4**). This is again in line with our previous finding in Syrian hamsters (Giroud et al., 2013) that showed an association, although weak (adjusted *R*^2^ of 0.04), between SR PL levels of DHA and SERCA2 activity. In this study, the higher strength (adjusted *R*^2^ of 0.33) of the relationship between SR PL levels of DHA and SERCA2 activity suggests that DHA has a stronger influence on SERCA2 activity, hence on T_b_ reached by the animals during torpor, than initially thought. Interestingly, torpid garden dormice in mid-hibernation, regardless of the dietary treatment, showed relatively high levels (∼21–28%) of DHA in their SR PL (**Table 6**). Indeed, previous studies reported much lower DHA levels in SR PL of alpine marmots (2.4%, Arnold et al., 2011) and Syrian hamsters (9.5%, Giroud et al., 2013). Further, levels of SERCA2 activity were about 3.5-times higher in garden dormice compared to those of hibernating Syrian hamsters at the same DHA concentration (Figure 4B in Giroud et al., 2013). The higher DHA proportions in SR PL of the insectivorous garden dormice might be linked to the fact that consumption of insects results in the intake of larger amounts of alpha linolenic acid (C18:3 n-3), permitting the synthesis and the incorporation of larger amounts of DHA into PL membranes. Further, various relative proportions of fatty acids can be incorporated into the different types of PLs. For instance, DHA could be incorporated into PLs that have the less interaction with SERCA proteins, hence the lowest influence on SERCA activity. Indeed, it has been shown that SERCA1a activity in skeletal muscles was regulated by the contents of both cholesterol and phosphatidylethanolamine (PE) in the membrane (Yeagle, 1989). Hence, garden dormice fed diets rich in n-3 PUFA had to incorporate lower amounts of DHA into lipids of which the composition is known to affect SERCA activity, such as PE or cholesterol esters, and higher proportions in other PLs, in order to tolerate significantly higher levels of DHA in their PL membranes compared to the more herbivorous and granivorous alpine marmots or Syrian hamsters.

## Conclusion

We demonstrated that high levels of DHA (and long-chain n-3 PUFA) in WAT, and most likely in cardiac SR PL, constrained dormice to delay their onset of hibernation. High levels of DHA might have dampened SERCA2 activity in the heart, preventing animals to maintain cardiac function at low T_b_ and hence to enter into torpor. We conclude that high DHA levels prevent hibernators from entering into torpor, but the critical levels differ substantially between species. Our data further support the occurrence of membrane remodeling during hibernation, with specific removal and/or oxidation of DHA, and retention of LA in tissues and membranes. Further studies would need to determine the underlying mechanisms and constraints of membrane remodeling during winter hibernation.

## Data Availability

The raw data supporting the conclusions of this manuscript will be made available by the authors, without undue reservation, to any qualified researcher.

## Author Contributions

SG and TR conceived and designed the study and analyzed the data. SG, GS, HG, AK-H, and JK performed the experiments. SG, TR, and WA interpreted the data. SG wrote the paper. All co-authors commented on the manuscript.

## Conflict of Interest Statement

JK is currently employed at International Flavors & Fragrances, Inc. During the time of the experiments and samples analyses, he was employed at the University of Veterinary Medicine Vienna. The remaining authors declare that the research was conducted in the absence of any commercial or financial relationships that could be construed as a potential conflict of interest.

## Footnotes

1 http://www.mpbio.com
