# Supplementary material for: Dietary Lipids Affect the Onset of Hibernation in the Garden Dormouse (Eliomys quercinus): Implications for Cardiac Function
Source: Front Physiol. 2018 Sep 18;9:1235. doi: 10.3389/fphys.2018.01235 (PMC6153335; doi:10.3389/fphys.2018.01235)
Supplement: Supplementary file 1 [file Table_1.docx]

Table S1: Parameters of linear models for the effects of diet treatment (‘Diet’) and sex (‘Sex’) on body masses at the start of the diet treatments (‘Pre-diet BM’) and prior to hibernation (‘Pre-hibernation BM’), as well as on energy intake during the diet treatments of garden dormice fed diets enriched with either n-6 fatty acids or n-3 fatty acids.

| **Variables** | **Diet** | | **Sex** | |
| --- | --- | --- | --- | --- |
|  | **t-statistic** | **p-value** | **t-statistic** | **p-value** |
|  |  |  |  |  |
| Pre-diet BM | -0.56 | 0.58 | 3.59 | *<0.01* |
| Pre-hibernation BM | -0.75 | 0.46 | 0.81 | 0.43 |
| Energy intake | -1.45 | 0.16 | -2.58 | *0.02* |

Table S2: Parameters of linear mixed-effects models with animal ID as random factor for the effects of diet treatment (‘Diet’) and time (‘Time’, pre-hibernation *vs.* mid-hibernation) on fatty acid proportions (% of total fatty acids) and ratios of certain fatty acid proportions from white adipose tissue total lipids of garden dormice fed diets enriched with either n-6 fatty acids or n-3 fatty acids. ‘PUFA’ refers to polyunsaturated fatty acids, ‘MUFA’ to monounsaturated fatty acids, ‘SFA’ to saturated fatty acids, ‘∑ n-6’ to the sum of n-6 PUFA, ‘∑ n-3’ to the sum of n-3 PUFA, and ‘n-6/n-3’ to the ratio between the sum of n-6 PUFA and the sum of n-3 PUFA. Significant p-values are shown in italic.

| **Fatty acids** | **Diet** | | **Time** | |
| --- | --- | --- | --- | --- |
|  | **t-statistic** | **p-value** | **t-statistic** | **p-value** |
|  |  |  |  |  |
| C14:0 | 7.49 | *<0.001* | 1.29 | 0.23 |
| C15:0 | 0.77 | 0.50 | 8.52 | *<0.001* |
| C16:0 | 4.45 | *0.003* | 14.78 | *<0.001* |
| C16:1 (n-7) | 5.96 | *<0.001* | 9.63 | *<0.001* |
| C17:0 | 2.40 | *0.04* | 7.87 | *<0.001* |
| C18:0 | 3.60 | *0.002* | 3.14 | *0.007* |
| C18:1 (n-9) | 0.98 | 0.40 | -0.41 | 0.69 |
| C18:2 (n-6) | -6.12 | *<0.001* | -17.11 | *<0.001* |
| C18:3 (n-3) | -0.02 | 0.99 | 2.61 | *0.02* |
| C20:4 (n-6) | 0.61 | 0.58 | 6.08 | *<0.001* |
| C20:5 (n-3) | 5.99 | *<0.001* | 6.18 | *<0.001* |
| C22:5 (n-3) | 5.61 | *<0.001* | 7.26 | *<0.001* |
| C22:6 (n-3) | 5.59 | *<0.001* | 6.56 | *<0.001* |
| PUFA | -3.97 | *<0.001* | -12.15 | *<0.001* |
| MUFA | 1.70 | 0.13 | 2.54 | *0.02* |
| SFA | 5.35 | *0.001* | 11.49 | *<0.001* |
| ∑ n-6 | -6.08 | *<0.001* | -17.41 | *<0.001* |
| ∑ n-3 | 4.75 | *<0.001* | 5.67 | *<0.001* |
| n-6/n-3 | -8.28 | *<0.001* | -11.88 | *<0.001* |
